# A particle swarm optimization algorithm based on an improved deb criterion for constrained optimization problems

**DOI:** 10.7717/peerj-cs.1178

**Published:** 2022-12-12

**Authors:** Ying Sun, Wanyuan Shi, Yuelin Gao

**Affiliations:** 1 North Minzu University, Collaborative Innovation Center of Scientific Computing and Intelligent Processing in Ningxia, Yinchuan, Ningxia, China; 2 North Minzu University, School of Mathematics and Information Sciences, Yinchuan, Ningxia, China

**Keywords:** Particle swarm optimization algorithm, Constrained optimization problems, Deb criterion

## Abstract

To solve the nonlinear constrained optimization problem, a particle swarm optimization algorithm based on the improved Deb criterion (CPSO) is proposed. Based on the Deb criterion, the algorithm retains the information of ‘excellent’ infeasible solutions. The algorithm uses this information to escape from the local best solution and quickly converge to the global best solution. Additionally, to further improve the global search ability of the algorithm, the DE strategy is used to optimize the personal best position of the particle, which speeds up the convergence speed of the algorithm. The performance of our method was tested on 24 benchmark problems from IEEE CEC2006 and three real-world constraint optimization problems from CEC2020. The simulation results show that the CPSO algorithm is effective.

## Introduction

Various types of constraints in numerous practical problems increase the difficulty of optimization. Problems with constraints are referred to as constrained optimization problems (COPs) (Sun & Yuan, 2006). Because the efficient solution of COPs is an important research topic in the optimization field, studying methods for solving COPs is both theoretically and practically important.

The core of the solution method for a COP is the constraint-handling technique employed. The key to designing a constraint-handling technique is balancing the objective function against the constraint function. The most popular constraint-handling techniques fall within three categories: (1) penalty function-based methods; (2) methods based on a feasibility criterion (a Deb criterion); and (3) multiobjective optimization-based methods. For the first method, a penalty factor is introduced into the constraint function, which is then included in the objective function, thereby converting the COP into an unconstrained optimization problem. These methods include the static penalty function (Kulkarni & Tai, 2011), dynamic penalty function (Liu et al., 2016), and adaptive penalty function (Yu et al., 2010). These methods are simple and easy to implement and have therefore been widely used in numerous algorithms. However, selecting penalty factors, which directly affect the results, is very difficult. For the second approach, Deb proposed this criterion in 2000 (Deb, 2000) as a means of selecting between solutions: a feasible solution is always preferred to an infeasible solution; for two feasible solutions, the one with a smaller fitness value is retained; and for two infeasible solutions, the one with a lower level of constraint violation is retained. This method is easy to implement, has a high convergence speed and is therefore widely used in various algorithms for solving COPs (Wang et al., 2016; Sarker, Elsayed & Ray, 2014; Elsayed, Sarker & Mezura-Montes, 2014). However, this method overemphasizes the importance of feasibility: under certain conditions, infeasible solutions may be closer to the global optimum than feasible solutions. Regardless, these ‘excellent’ infeasible solutions containing useful information would be discarded using the Deb criterion. Thus, studying how to better utilize these ‘excellent’ infeasible solutions is necessary. For the third problem, a COP is transformed into an unconstrained multiobjective optimization problem with two objectives (Wang et al., 2007): the objective function of the original problem and a constraint-violation-degree function composed of all the constraint functions of the original problem. This method can be solved by existing multiobjective algorithms (Wang, Dai & Hu, 2010; Cai, Hu & Fan, 2013), and it can produce a good diversity of solutions. However, multiobjective optimization problems have a high computational time complexity and are difficult to solve.

With the rapid development of intelligent algorithms, different algorithms have been integrated into the abovementioned methods for solving COPs. For example, Wang et al. (2016) proposed a FROFI algorithm in which feasibility criteria were incorporated into a differential evolution (DE) algorithm to solve COPs. Karaboga & Akay (2011) integrated the Deb criterion and an improved artificial bee colony (ABC) algorithm and used a probabilistic selection scheme for feasible solutions based on their fitness values. Francisco, Costa & Rocha (2016) combined the firefly algorithm with feasibility and dominance rules and a fitness function based on global competitive ranking to solve nonsmooth nonconvex constrained global optimization problems. Kohli & Arora (2018) proposed the chaotic grey wolf algorithm for COPs. Kimbrough et al. (2008) used a data-driven approach to analyse the dynamics of a two-population genetic algorithm (GA). Among numerous available algorithms, the particle swarm optimization (PSO) algorithm proposed by Kennedy & Eberhart (1995) has a small number of parameters and a fast convergence and has therefore attracted considerable attention for solving COPs. Yadav & Deep (2014) proposed a coswarm PSO algorithm for nonlinear constrained optimization. In this algorithm, the total swarm is subdivided into two sub swarms. The first swarm uses the shrinking hypersphere PSO (SHPSO), and the second uses DE. Venter & Haftka (2010) converted the COP into an unconstrained biobjective optimization, which was solved by a multiobjective PSO algorithm. Liu et al. (2018) proposed a parallel boundary search PSO algorithm, in which an improved constrained PSO algorithm is adopted to conduct a global search for one branch, while the subset constrained boundary narrower (SCBN) function and sequential quadratic programming (SQP) are applied to perform a local boundary search for a different branch. A cooperative mechanism of the two branches guides the global search direction to the boundaries of the active constraints. Liu, Cai & Wang (2010) integrated the PSO algorithm with the DE algorithm to solve COPs. When the PSO algorithm remains stagnant, introducing DE during the PSO update forces the PSO algorithm out of stagnation. Wang & Cai (2009) used a parallel search operator to divide an existing swarm into multiple subspaces and a local PSO algorithm as the search engine for each subswarm. DE improves the global search by evolving the optimal solution for individual particles. Kohler, Vellasco & Tanscheit (2019) proposed a novel PSO algorithm, PSO+, which uses a feasibility repair operator and two swarms to ensure that a swarm always exists with particles that fully satisfy every constraint. In addition, diversity is inserted into the swarm to improve the search-space coverage. Ang et al. (2020) introduced a novel constraint-handling technique to guide the population search towards the feasible regions of the search space. Two evolution phases, known as current swarm evolution and memory swarm evolution, are introduced to provide multiple search operators for individual particles and thereby improve the robustness of the algorithm for solving different types of COPs.

A PSO algorithm based on an improved Deb criterion was developed in this study to solve COPs. Useful information from ‘excellent’ infeasible solutions is retained to guide the algorithm in jumping out of a local extremum and to accelerate convergence to the global optimal solution. Moreover, inspired by the use of HMPSO (Wang & Cai, 2009) and PSO-DE (Liu, Cai & Wang, 2010) to improve the global search ability, DE was introduced to optimize the particle positions in the swarm, thereby increasing the convergence speed of the proposed algorithm.

## Introduction to cops

A general COP can be expressed as follows (Sun & Yuan, 2006):

(1) $$\eqalign{& \min {\rm }f(x) \cr & s.t.\ {\rm }{g_j}(x) \le 0,j = 1,2, {\rm \bf L } ,q \cr & {\rm }{h_j}(x) = 0,j = q + 1,q + 2, {\bf L} ,m \cr & {\rm }x = ({x_1},{x_2}, {\rm \bf L } ,{x_D}) \in S \cr & {\rm }{L_i} \le {x_i} \le {U_i},i = 1,2, {\rm \bf L } ,D}$$

where $x \in S$ is the decision variable; $S = \prod\nolimits_{i = 1}^D {[{L_i},{U_i}]}$ is the decision space; ${L_i}$ and ${U_i}$ are the lower and upper bounds of the *i*th component, respectively; $f(x)$ is the objective function; ${g_j}(x)$ is the *j*th inequality constraint; ${h_j}(x)$ is the *j*th equality constraint; and $q$ and $m - q$ are the numbers of inequality and equality constraints, respectively.

For a COP, the degree of violation of each decision vector $x$ for the *j*th constraint is defined as

${G_j}(x) = \left\{ \matrix{\max (0,{g_j}(x)), & 1 \le j \le q \hfill \cr \max (0,|{h_j}(x)| - \delta ) & q + 1 \le j \le m \hfill} \right.{\rm }$

where $\delta$ is the slack in the equality constraint. Therefore, the degree of violation of the decision vector *x* for all constraints can be defined as given below.

$G(x) = \sum\limits_{j = 1}^m {{G_j}(x)}$

If the degree of constraint violation of the decision vector *x* is $G(x) = 0$, then the decision vector is a feasible solution of the COP; otherwise, it is an infeasible solution. The feasible solution with the minimum value is the optimal feasible solution. Thus, the goal of solving the COP is to find the feasible solution with the minimum function value.

## A pso algorithm based on an improved deb criterion

PSO is an evolutionary technique that was proposed by Dr. Eberhart and Dr. Kennedy in 1995 (Kennedy & Eberhart, 1995). PSO originated from research on the predatory behaviour of birds. PSO is similar to the genetic algorithm (GA) in that it is an iterative-based optimization method; that is, a system is initialized using a set of random solutions, and the optimal value is then searched through iteration.

**(1) Classical PSO algorithm**

Consider an n-dimensional target search space containing a population of N particles, each of which is regarded as a point. Each particle is characterized by a unique position vector $x = ({x_1},{x_2},...,{x_n})$ that corresponds to a different fitness function value of the objective function.

**Algorithm 3.1 Adaptive PSO (APSO)**

**Step 1**: Initialization. The population size *N*, self-cognitive coefficient ${c_1}$, and social-cognitive coefficient ${c_2}$ are determined. The *i*-th particle ( $i = (1,2,...,N)$) in the *n-*dimensional space is characterized by a position vector ${x_i}$ and a velocity vector ${v_i}$. The maximum number of iterations is ${T_{\max }}$. An initial swarm $X(0)$ of *N* particles is randomly generated, and $t: = 0$ is set.

**Step 2:** Particle evaluation. The fitness value of each particle is calculated or evaluated.

**Step 3:** The velocity and position of each particle are updated using the following equations:

(2) $$v_{id}^{T + 1} = wv_{id}^T + {c_1}{r_1}({p_{ibest}}_d - x_{id}^T) + {c_2}{r_2}({g_{best}}_d - x_{id}^T)$$

(3) $$x_{id}^{T + 1} = x_{id}^T + v_{id}^{T + 1}$$

(4) $$w = {w_{\max }} - \displaystyle{{t({w_{\max }} - {w_{\min }})} \over {{T_{\max }}}}$$

where ${w_{\max }}$ and ${w_{\min }}$ are the maximum and minimum values of the inertia weights, respectively.

**Step 4:** The best position ${p_{ibest}}$ of the $i {-} {\rm th}$ particle and the global best position ${g_{best}}$ are updated for each particle.

**Step 5:** Termination. If the termination Deb criterion is met, the global optimal value is output as the optimal solution, and the calculation process is terminated. Otherwise, $t: = t + 1$ is set, and the process is repeated from Step 2.

Unlike the typical PSO algorithm, the APSO algorithm introduces an adaptive linear decreasing function into the selection of inertia weights, which is similar to the concept of a global search.

**(2) Improved Deb criterion**

**Figure 1:**
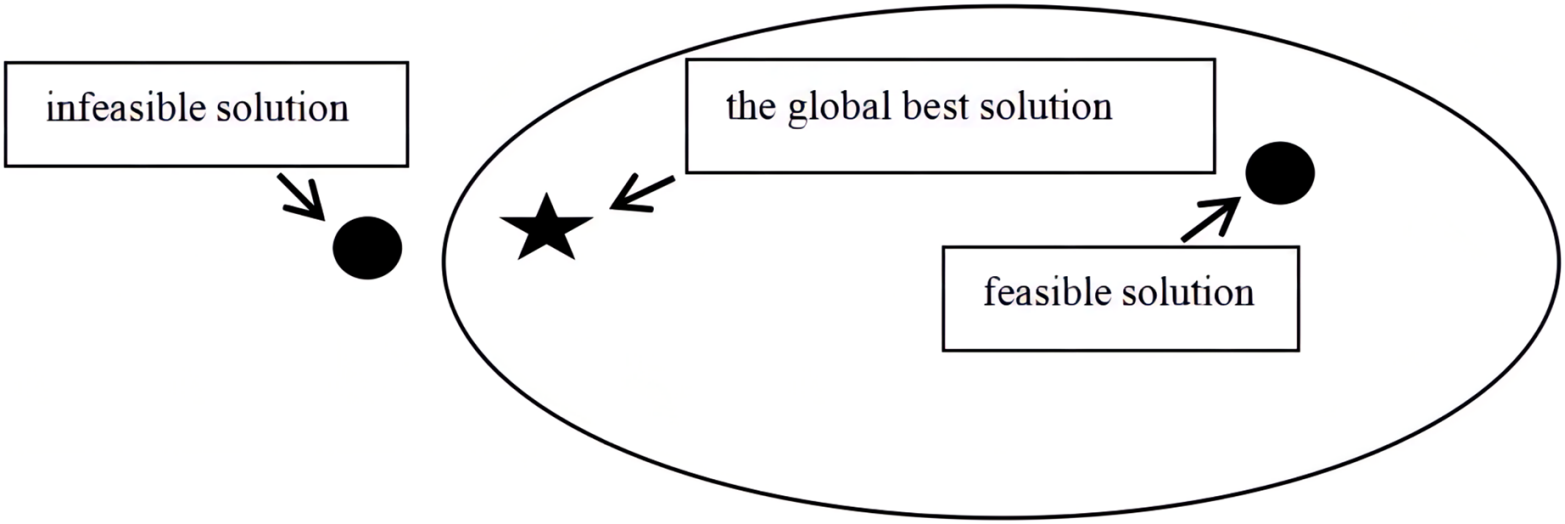
The position relation of feasible solution, infeasible solution and global best solution.

Unlike the methods used to solve general unconstrained optimization problems, the entire space must be searched to solve COPs. Having to both optimize the objective function and ensure the feasibility of the solution during the evolution process inevitably increases the difficulty of the solution procedure. Hence, the development of the Deb criterion has attracted considerable attention. To satisfy the Deb criterion, a feasible solution is used to replace an infeasible solution to become the optimal particle position for the current generation. However, the condition shown in Fig. 1 often occurs, that is, the infeasible solution is closer to the global best position than the feasible solution. Discarding the infeasible solution to satisfy the Deb criterion affects the convergence speed. Therefore, we propose an improved Deb criterion. Specifically, an ‘excellent’ infeasible solution for which the objective function values are close to the global best solution is stored. The rules for this procedure are given below.

- If two particles represent feasible solutions, the particle with the smaller fitness value is retained.
- If one particle represents a feasible solution and the other represents an infeasible solution, the particle representing the feasible solution is retained. Moreover, an infeasible solution with a small fitness value is stored in an ‘excellent’ infeasible solution set *A*.
- If both particles represent infeasible solutions, the particle with a lower degree of constraint violation is retained. Moreover, an infeasible solution for an unselected particle with a small fitness value is also stored in the ‘excellent’ infeasible solution set *A*.

Thus, an ‘excellent’ infeasible solution set is built from infeasible solutions with small fitness values. In subsequent operations, these particles are used to update the current swarm, where the information from these infeasible solutions is used in the iterative process.

**(3) Updating the optimal particle position**

After each iteration of the algorithm, the optimal particle position is updated using the improved Deb criterion. Because the PSO algorithm can easily fall into a local optimum, DE has been integrated into the PSO algorithm to improve the global search ability (Yadav & Deep, 2014; Liu, Cai & Wang, 2010; Wang & Cai, 2009) with good results. Therefore, DE was used to improve the global search ability of the algorithm in this study.

The following steps are performed.

(i) Randomly select ${r_1},{r_2} \in \{ 1,2,...,N\}$ for the optimal position of the *i*th particle, $i = 1,2,...,N$.
(ii) Mutation: ${v_i} = Pbes{t_i} + F(Pbes{t_{{r_2}}} - Pbes{t_{{r_1}}})$, where ${v_i}$ is an intermediate variable, and *F* is a scaling factor.
(iii) Cross operation: $for\,{\rm }j = 1{:}n$

${u_{i,j}} = \left\{ {\matrix{ {{v_{i,j}}} & {if\quad{\rm }ran{d_j} \le CR\ {\rm }or\ {\rm }j = {j_{rand}}} \cr {Pbes{t_{i,j}}} & {otherwise} \cr } } \right.$

where ${u_{i,j}}$ is an intermediate variable, $CR$ is the crossover probability, and ${j_{rand}}$ is a random integer, $\{ 1,2,...,n\}$.

(iv) If ${u_{i,j}}$ exceeds the upper and lower bounds of the *j*th dimension, the following equation is used (Liu, Cai & Wang, 2010):

${u_{i,j}} = \left\{ {\matrix{ {{L_j}} & {if\quad{\rm }rand \le 0.5{\rm \,and }\,{u_{i,j}} \lt {L_j}} \cr {2*{L_j} - {u_{i,j}}} & {if\quad{\rm }rand \gt 0.5\,{\rm and }\,{u_{i,j}} \lt {L_j}} \cr {{U_j}} & {if\quad{\rm }rand \le 0.5\,{\rm and }\,{u_{i,j}} \gt {U_j}} \cr {2*{U_j} - {u_{i,j}}} & {if\quad{\rm }rand \gt 0.5\,{\rm and }\,{u_{i,j}} \gt {U_j}} \cr } } \right.$

where $rand$ is a random number within [0, 1] with a normal distribution.

(v) Compare ${u_i}$ and $Pbes{t_i}$ based on the improved Deb criterion. If ${u_i}$ is better, then update $Pbes{t_i}$ and the infeasible solution set *A*.

Note that the DE strategy is incorporated into the HMPSO algorithm (Wang & Cai, 2009) to update the optimal positions of individual particles. However, the algorithm performs a mutation operation on a particle’s optimal position by using three other particles to generate an intermediate particle, without using the information for the optimal position of the particle to be mutated. The optimal position of a particle determined by the PSO algorithm is the best position of the particle over all iterations, which contains historical information and should not be directly discarded. Therefore, the optimal position of the particle to be mutated is retained, and the optimal positions of only two other particles are used in the mutation operation.

**(4) Updating the current swarm using the infeasible solution set**

The PSO algorithm differs from the DE and GA algorithms. Both of these algorithms only perform evolutionary optimization of the current population. However, the PSO algorithm stores both the current swarm and the optimal-particle swarm, that is, the optimal solutions of the particles since the first iteration. Many PSO modifications were developed based on the optimal particle swarm, and the effect of the efficient evolution of the current swarm on the entire algorithm was neglected. In “Classical PSO algorithm”, we used the improved Deb criterion to obtain an ‘excellent’ infeasible solution set *A*. Inspired by the FROFI algorithm (Wang et al., 2016), this infeasible solution set is used to update the current particle swarm. However, the FROFI algorithm only uses an external set to update one particle of the current swarm in each iteration, such that information from excellent infeasible solutions is not used. Hence, the infeasible solution set was used in this study to guide the evolution of the current swarm.

The following steps are performed.

- A nondominance operation is performed on the elements of set *A*; that is, the objective function value and the degree of constraint violation are regarded as two objective values. Particle dominance is considered as follows: if the objective function value and degree of constraint violation of a particle $a_1$ are higher than those of $a_2$, then $a_1$ is removed from the swarm. This process is repeated until all dominant particles are removed.
- The particle with the lowest degree of constraint violation in set *A* and the particle with the highest degree of constraint violation in the current swarm are compared. If the former degree is larger than the latter degree, the current particle is replaced with the former particle and deleted from set *A*. This process is repeated until the degree of constraint violation of the particle with the lowest degree of constraint violation in set *A* is larger than the highest degree of constraint violation in the current swarm.

**(5) Retention strategy used to determine the best global position**

The following retention strategy is used to determine the global best position. (i) The particles with global optimal positions and the optimal particle swarm are combined into a candidate swarm *U*. (ii) If swarm *U* contains feasible solutions, the particle solution with the smallest fitness value is stored as the global best position. If swarm *U* does not contain feasible solutions, the particle solution with the smallest constraint violation value is stored as the global best position.

**(6) PSO algorithm based on the improved Deb criterion**

**Algorithm 2**

The procedure for the PSO algorithm for constrained optimization problems (CPSO) is summarized below.

**Step 1:** Initialization. Randomly initialize the position vector $X = ({x_1},{x_2},...,{x_N})$ and the velocity vector $V = ({v_1},{v_2},...,{v_N})$; set the parameters ${c_1}$, ${c_2}$, ${w_{\max }}$, ${w_{\min }}$, $F$, $CR$, ${T_{\max }}$, $N$, and *D* (the dimensions of the decision vector); and set *t* = 0.

**Step 2:** Calculate the objective function value of each particle in the initial swarm, where the optimal positions of the initial particles are ${P_{best}} = X$. Store ${g_{best}}$ using the retention strategy described in “Updating the Current Swarm Using the Infeasible Solution Set”.

**Step 3:** Update the current swarm based on Eqs. (2) and (3) to generate a new generation ${x_{i,j}}^{t + 1}$, and use the following equation to treat out-of-bound particles (Liu et al., 2018).

${x_{i,j}}^{t + 1} = \left\{ {\matrix{ {0.5({x_{i,j}}^t + {L_j})} & {if\,\,{\rm }{x_{i,j}}^t \lt {L_j}} \cr {0.5({x_{i,j}}^t + {U_j})} & {if\,\,{\rm }{x_{i,j}}^t > {U_j}} \cr {{x_{i,j}}^{t + 1}} & {otherwise} \cr } } \right.;$

**Step 4**: Update the optimal particle positions ${p_{ibest}}$ and $i = (1,2,...,N)$ based on the improved Deb criterion proposed in “Classical PSO algorithm”, and store ‘excellent’ infeasible solutions in set *A*.

**Step 5**: Use DE (as described in “Improved Deb criterion”) to update the optimal particle position and set *A*.

**Step 6**: Update the current swarm using set *A*, and after the update, let $A = \phi$.

**Step 7**: Update ${g_{best}}$ based on the retention strategy.

**Step 8:** Termination. If the termination criterion is met, the global best particle is output as the optimal solution, and the calculation process is terminated. Otherwise, let $t: = t + 1$, and return to Step 2.

**(7) Complexity Analysis**

We present the computational time complexity of the CPSO algorithm below, considering only the worst-case scenarios for the main steps during one iteration for a swarm of size *N*.

1. Updating a particle: $O(N)$
2. Updating the optimal particle position and the set *A*: $O(N)$
3. DE updating strategy: $O(N)$
4. Update the current swarm using set *A* (for the worst case in which all the current particles must be replaced): $O(N\log N)$
5. The retention strategy for the global best position: $O(N)$

Thus, the computational time complexity of the CPSO algorithm for the worst case is $O(N\log N)$, which demonstrates that the algorithm is computationally efficient.

## Test functions and parameter settings

A total of 24 test functions from IEEE CEC2006 (Liang et al., 2006) were used to further evaluate the performance of the proposed algorithm. However, because finding feasible solutions for functions G20 and G22 is widely believed to be difficult, they were subsequently excluded. The 22 remaining functions were of various types. Table 1 shows the specific characteristics of the functions. *N* represents the number of decision variables. Linear, nonlinear, polynomial, quadratic, and cubic objective functions are considered. $\rho = |\Omega |/|S|$ is the estimated ratio between the randomly simulated feasible area and the search space, $|S|$ is the number of randomly generated solutions in the search space, and $|\Omega |$ is the number of feasible solutions in $|S|$. The number of simulations is usually 1,000,000. *LI* and *NI* represent linear and nonlinear inequality constraints, respectively. *LE* and *NE* represent linear and nonlinear equality constraints, respectively. *a* is the number of active constraint functions near the optimal solution. The optimal solutions of the considered functions are known, where $f({x^*})$ denotes the global optimal function value. The improved optimal solution of G17 obtained in the literature (Wang & Cai, 2011) was used in this study.

**Table 1:** Salient features of 22 test problems.

*Prob*.	*n*	*Type of objective function*	$\rho$ (%)	*LI*	*NI*	*LE*	*NE*	$a$	$f(x^{*})$
G01	13	Quadratic	0.0111%	9	0	0	0	6	−15.0000000000
G02	20	Nonlinear	99.9971%	0	2	0	0	1	−0.8036191042
G03	10	Polynomial	0.0000%	0	0	0	1	1	−1.0005001000
G04	5	Quadratic	52.1230%	0	6	0	0	2	−30,665.5386717834
G05	4	Cubic	0.0000%	2	0	0	3	3	5,126.4967140071
G06	2	Cubic	0.0066%	0	2	0	0	2	−6,961.8138755802
G07	10	Quadratic	0.0003%	3	4	0	0	6	24.3062090681
G08	2	Nonlinear	0.8560%	0	2	0	0	0	−0.0958250415
G09	7	Polynomial	0.5121%	0	4	0	0	2	680.6300573745
G10	8	Linear	0.0010%	3	3	0	0	6	7,049.2480205286
G11	2	Quadratic	0.0000%	0	0	0	1	1	0.7499000000
G12	3	Quadratic	4.7713%	0	1	0	0	0	−1.0000000000
G13	5	Nonlinear	0.0000%	0	0	0	3	3	0.0539415140
G14	10	Nonlinear	0.0000%	0	0	3	0	3	−47.7648884595
G15	3	Quadratic	0.0000%	0	0	1	1	2	961.7150222899
G16	5	Nonlinear	0.0204%	4	34	0	0	4	−1.9051552586
G17	6	Nonlinear	0.0000%	0	0	0	4	4	8,853.53387480648
G18	9	Quadratic	0.0000%	0	13	0	0	6	−0.8660254038
G19	15	Nonlinear	33.4761%	0	5	0	0	0	32.6555929502
G21	7	Linear	0.0000%	0	1	0	5	6	193.7245100700
G23	9	Linear	0.0000%	0	2	3	1	6	−400.0551000000
G24	2	Linear	79.6556%	0	2	0	0	2	−5.5080132716

To compare the performances of different algorithms, the parameters of HMPSO (Wang & Cai, 2009) were used for CPSO: ${c_1} = 1.7$, ${c_2} = 1.7$, ${w_{\max }} = 0.9$, ${w_{\min }} = 0.5$, $F = 0.7$, $CR = 1.0$, number of evaluations of the fitness function = $5 \times {10^5}$, and $\delta = 0.0001$. The algorithm was programmed and run in MATLAB software using a computer with an Intel (R) core (TM) i7-6567U CPU @ 3.30 GHz 3.20 GHz processor and 8.00 GB of memory.

The Wilcoxon rank-sum test was used to compare the performance of these algorithms. The null hypothesis is that there is no significant difference in the performance between the proposed algorithm and the corresponding algorithm. The symbol (+) indicates that the proposed algorithm is significantly better compared to the corresponding algorithms based on the Wilcoxon rank-sum test at the α = 0.05 significance level; the symbol (−) indicates significantly worse, and the symbol (=) indicates no significant difference (Derrac et al., 2011).

## Results

(1) Comparison of improved PSO algorithms based on different strategies

We introduced the improved Deb criterion (IDeb) and the DE update strategy into the classic PSO algorithm. To demonstrate the effectiveness of the individual strategies, two algorithms were constructed, *i.e*., PSO+Deb+DE and PSO+IDeb. The same parameters were used for CPSO, PSO+Deb+DE, and PSO+IDeb to facilitate comparison of the numerical results.

Table 2 shows the feasible rate and success rate of CPSO, PSO+Deb+DE and PSO+IDeb for the 22 test functions after 25 runs. The *feasible rate* refers to the proportion of feasible solutions in the numerical results, and the *success rate* refers to the proportion of solutions for which the error in the function value is $f(x) - f({x^*}) \le 0.0001$, where *x* is a feasible solution. The formula is as follows:

**Table 2:** The feasible rate and success rate of the three algorithms on 22 test problems.

Prob.	Feasible rate%			Success rate%		
	CPSO	PSO+Deb+DE	PSO+IDeb	CPSO	PSO+Deb+DE	PSO+IDeb
G01	100	100	100	100	88	8
G02	100	100	100	36	0	0
G03	100	100	100	96	92	0
G04	100	100	100	100	100	100
G05	100	100	100	100	100	8
G06	100	100	100	100	100	100
G07	100	100	100	100	100	0
G08	100	100	100	100	100	100
G09	100	100	100	100	100	100
G10	100	100	100	100	100	0
G11	100	100	100	100	100	100
G12	100	100	100	100	100	100
G13	100	100	100	48	40	0
G14	100	100	100	100	100	0
G15	100	100	100	100	100	44
G16	100	100	100	100	100	100
G17	100	100	100	36	32	0
G18	100	100	100	100	100	16
G19	100	100	100	100	100	0
G21	96	92	20	52	64	0
G23	100	100	64	100	96	0
G24	100	100	100	100	100	100

$feasible\,rate = \displaystyle{{{A^*}} \over A}$

$success\,rate = \displaystyle{{{A^{**}}} \over {{A^*}}}$

where ${A^*}$ denotes the number of feasible solutions obtained by the algorithm, $A$ denotes the number of solutions obtained by the algorithm, ${A^{**}}$ denotes the number of solutions for which the error in the function value $f(x) - f({x^*}) \le 0.0001$, *x* is a feasible solution, and ${x^*}$ is the known optimal solution.

The numerical results show that the feasible rates of CPSO and PSO+Deb+DE reached 100%, except for G21. The feasible rate of CPSO was 96% for G21 (that is, one out of 25 runs converged outside the feasible region), which was higher than that for PSO+Deb+DE (92%) and PSO+IDeb (20%). PSO+IDeb had a lower feasible rate of 64% for G23. A 100% success rate was obtained for 17 functions by CPSO, for 15 functions by PSO+Deb+DE, and for eight functions by PSO+IDeb. After 25 runs, PSO+Deb+DE had not successfully solved one function (G02) compared to 10 functions for PSO+IDeb. These results show that introducing DE to update the optimal particle solution set increased the feasible rate of the PSO algorithm and improved the global search ability. In particular, the feasible rates of all three algorithms reached 100% for G02, but the success rates were 32%, 0%, and 0%. Thus, the feasible space of a function can be easily found, but the global best solution is difficult to obtain. The success rate of PSO+IDeb was not high but represented an improvement over that of PSO.

The abovementioned numerical results were analysed in detail. Table 3 shows the numerical results obtained using the three algorithms for the 22 test functions after 25 runs. In the table, *Best* denotes the best solution, *Worst* denotes the worst solution, *Median* denotes the median value, *Mean* denotes the mean value, and *Std* denotes the standard deviation. *Wil test* denotes the Wilcoxon test result. The success rates show that the functions for which a success rate of 100% was obtained were relatively stable, with a small standard deviation. Next, the five functions for which the CPSO success rate did not reach 100% (*i.e*., G02, G03, G13, G17, and G21) were considered. (a) For G02, only CPSO obtained the optimal solution of −8.036191E−01. The *Std* obtained using CPSO was also the smallest, indicating that the algorithm results fluctuated around the optimal solution. (b) For G03, the results of CPSO and PSO+Deb+DE were close, although a smaller *Std* was obtained by using CPSO than by using PSO+Deb+DE. By comparison, PSO+IDeb did not produce good results. (c) For G13, CPSO and PSO+Deb+DE produced very close and good results, whereas the optimal value obtained by PSO+IDeb was far from the best known solution and indicated a nonideal performance. (d) For G17, both CPSO and PSO+Deb+DE obtained the most recently obtained optimal solution (8,853.53387480648), where the *mean* of CPSO was closer to the global best solution, but the *std* of PSO+Deb+DE was smaller. In addition, the PSO+IDeb result was some distance from the latest optimal solution. (e) For G21, the stability of the three algorithms was comparable, although that of CPSO was slightly superior. Both CPSO and PSO+Deb+DE obtained the global best solution. However, none of the three algorithms solved the problem stably. The results presented in Tables 2 and 3 show that CPSO was superior to the other two algorithms for solving the 22 functions; that is, the integration of Deb and DE improved the ability of the PSO algorithm to solve the COPs. The results of the Wilcoxon test show that the performance of CPSO is significantly better than PSO+IDeb, which is equivalent to PSO+Deb+DE. However, combined with the success rates, the strategy of IDeb and DE can be used simultaneously to solve these 22 problems.

**Table 3:** Results obtained by the three algorithms on 22 test problems.

Prob.	Algorithm	Best	Worst	Median	Mean (Wil Test)	Std
G01	CPSO	−1.500000E+01	−1.500000E+01	−1.500000E+01	−1.500000E+01	0
	PSO+Deb+DE	−1.500000E+01	−1.300000E+01	−1.500000E+01	−1.476000E+01(=)	6.6333E−01
	PSO+IDeb	−1.500000E+01	−1.450557E−21	−6.000000E+00	−7.832492E+00(+)	4.2253E+00
G02	CPSO	−8.036191E−01	−7.485572E−01	−7.948968E−01	−7.872680E−01	1.9431E−02
	PSO+Deb+DE	−7.852643E−01	−5.323286E−01	−7.425712E−01	−7.192754E−01(+)	6.9977E−02
	PSO+IDeb	−8.023317E−01	−3.380794E−01	−6.101827E−01	−6.145027E−01(+)	1.1817E−01
G03	CPSO	−1.000500E+00	−9.945233E−01	−1.000500E+00	−1.000261E+00	1.1953E−03
	PSO+Deb+DE	−1.000500E+00	−9.694231E−01	−1.000500E+00	−9.990905E−01(=)	6.2359E−03
	PSO+IDeb	−9.181721E−01	−9.532822E−04	−3.325069E−01	−3.516404E−01(+)	3.5625E−02
G04	CPSO	−3.066554E+04	−3.066554E+04	−3.066554E+04	−3.066554E+04	3.7130E−12
	PSO+Deb+DE	−3.066554E+04	−3.066554E+04	−3.066554E+04	−3.066554E+04(=)	3.7130E−12
	PSO+IDeb	−3.066554E+04	−3.066554E+04	−3.066554E+04	−3.066554E+04(=)	3.7130E−12
G05	CPSO	5.126497E+03	5.126497E+03	5.126497E+03	5.126497E+03	2.7847E−12
	PSO+Deb+DE	5.126497E+03	5.126497E+03	5.126497E+03	5.126497E+03(=)	2.7847E−12
	PSO+IDeb	5.126497E+03	6.095104E+03	5.169455E+03	5.544048E+03(+)	4.4784E+02
G06	CPSO	−6.961814E+03	−6.961814E+03	−6.961814E+03	−6.961814E+03	0
	PSO+Deb+DE	−6.961814E+03	−6.961814E+03	−6.961814E+03	−6.961814E+03(=)	0
	PSO+IDeb	−6.961814E+03	−6.961814E+03	−6.961814E+03	−6.961814E+03(=)	0
G07	CPSO	2.430621E+01	2.430621E+01	2.430621E+01	2.430621E+01	8.2685E−15
	PSO+Deb+DE	2.430621E+01	2.430621E+01	2.430621E+01	2.430621E+01(=)	1.7896E−14
	PSO+IDeb	2.438534E+01	1.830037E+02	2.515368E+01	3.181996E+01(+)	3.3653E+01
G08	CPSO	−9.582504E−02	−9.582504E−02	−9.582504E−02	−9.582504E−02	1.2981E−17
	PSO+Deb+DE	−9.582504E−02	−9.582504E−02	−9.582504E−02	−9.582504E−02(=)	1.3878E−17
	PSO+IDeb	−9.582504E−02	−9.582504E−02	−9.582504E−02	−9.582504E−02(=)	4.0062E−18
G09	CPSO	6.806301E+02	6.806301E+02	6.806301E+02	6.806301E+02	2.3206E−13
	PSO+Deb+DE	6.806301E+02	6.806301E+02	6.806301E+02	6.806301E+02(=)	2.3206E−13
	PSO+IDeb	6.806301E+02	6.806301E+02	6.806301E+02	6.806301E+02(=)	9.4636E−05
G10	CPSO	7.049248E+03	7.049248E+03	7.049248E+03	7.049248E+03	2.9646E−12
	PSO+Deb+DE	7.049248E+03	7.049248E+03	7.049248E+03	7.049248E+03(=)	3.1506E−12
	PSO+IDeb	7.281934E+03	8.487416E+03	7.891132E+03	7.786813E+03(+)	4.6290E+02
G11	CPSO	7.499000E−01	7.499000E−01	7.499000E−01	7.499000E−01	1.1331E−16
	PSO+Deb+DE	7.499000E−01	7.499000E−01	7.499000E−01	7.499000E−01(=)	1.1331E−16
	PSO+IDeb	7.499000E−01	7.499000E−01	7.499000E−01	7.499000E−01(=)	1.1102E−16
G12	CPSO	−1.000000E+00	−1.000000E+00	−1.000000E+00	−1.000000E+00	0
	PSO+Deb+DE	−1.000000E+00	−1.000000E+00	−1.000000E+00	−1.000000E+00(=)	0
	PSO+IDeb	−1.000000E+00	−1.000000E+00	−1.000000E+00	−1.000000E+00(=)	0
G13	CPSO	5.394151E−02	4.388026E−01	4.388026E−01	2.540693E−01	1.9624E−01
	PSO+Deb+DE	5.394151E−02	1.000000E+00	4.388026E−01	3.522019E−01(=)	3.0540E−01
	PSO+IDeb	7.436901E−02	2.520161E+00	7.870618E−01	7.766994E−01(+)	6.3030E−01
G14	CPSO	−4.776489E+01	−4.776489E+01	−4.776489E+01	−4.776489E+01	2.9008E−14
	PSO+Deb+DE	−4.776489E+01	−4.776489E+01	−4.776489E+01	−4.776489E+01(=)	2.9970E−14
	PSO+IDeb	−4.751686E+01	−3.522036E+01	−4.250627E+01	−4.194900E+01(+)	3.6848E+00
G15	CPSO	9.617150E+02	9.617150E+02	9.617150E+02	9.617150E+02	5.8016E−13
	PSO+Deb+DE	9.617150E+02	9.617150E+02	9.617150E+02	9.617150E+02(=)	5.8016E−13
	PSO+IDeb	9.617150E+02	9.714031E+02	9.617327E+02	9.642034E+02(+)	3.5206E+00
G16	CPSO	−1.905155E+00	−1.905155E+00	−1.905155E+00	−1.905155E+00	4.5325E−16
	PSO+Deb+DE	−1.905155E+00	−1.905155E+00	−1.905155E+00	−1.905155E+00(=)	4.5325E−16
	PSO+IDeb	−1.905155E+00	−1.905112E+00	−1.905155E+00	−1.905152E+00(+)	9.4800E−02
G17	CPSO	8.853534E+03	8.927979E+03	8.927592E+03	8.900967E+03	3.6308E+01
	PSO+Deb+DE	8.853534E+03	8.928355E+03	8.927592E+03	8.903943E+03(=)	3.5294E+01
	PSO+IDeb	8.862857E+03	9.278930E+03	9.011912E+03	9.040134E+03(+)	1.2451E+02
G18	CPSO	−8.660254E−01	−8.660254E−01	−8.660254E−01	−8.660254E−01	4.5325E−17
	PSO+Deb+DE	−8.660254E−01	−8.660254E−01	−8.660254E−01	−8.660254E−01(=)	2.2888E−16
	PSO+IDeb	−8.660163E−01	−4.991166E−01	−8.641657E−01	−7.940803E−01(+)	1.1888E−01
G19	CPSO	3.265559E+01	3.265559E+01	3.265559E+01	3.265559E+01	2.2328E−14
	PSO+Deb+DE	3.265559E+01	3.265559E+01	3.265559E+01	3.265559E+01(=)	2.5905E−14
	PSO+IDeb	3.393308E+01	9.548221E+01	5.001483E+01	5.448720E+01(+)	2.6790E+01
G21	CPSO	1.937245E+02	9.454397E+02	1.937245E+02	2.657074E+02	1.5449E+02
	PSO+Deb+DE	1.937245E+02	9.618374E+02	1.937245E+02	2.897845E+02(=)	2.0339E+02
	PSO+IDeb	2.557580E+02	9.999908E+02	8.620895E+02	7.866938E+02(+)	2.7833E+02
G23	CPSO	−4.000551E+02	−4.000550E+02	−4.000551E+02	−4.000551E+02	1.5844E−05
	PSO+Deb+DE	−4.000551E+02	−4.000487E+02	−4.000551E+02	−4.000548E+02(=)	1.2780E−03
	PSO+IDeb	−2.100001E+03	6.835154E+02	−3.900000E−03	−3.350658E+02(+)	8.2946E+02
G24	CPSO	−5.508013E+00	−5.508013E+00	−5.508013E+00	−5.508013E+00	9.0649E−16
	PSO+Deb+DE	−5.508013E+00	−5.508013E+00	−5.508013E+00	−5.508013E+00(=)	9.0649E−16
	PSO+IDeb	−5.508013E+00	−5.508013E+00	−5.508013E+00	−5.508013E+00(=)	9.0649E−16
+/=/—:		PSO+Deb+DE	1/21/0	PSO+IDeb	15/7/0	

**Note:**

Best denotes the best solution; Worst denotes the worst solution; Median denotes the median value; Mean denotes the mean value; and Std denotes the standard deviation; Wil test denotes the Wilcoxon test result.

**Figure 2:**
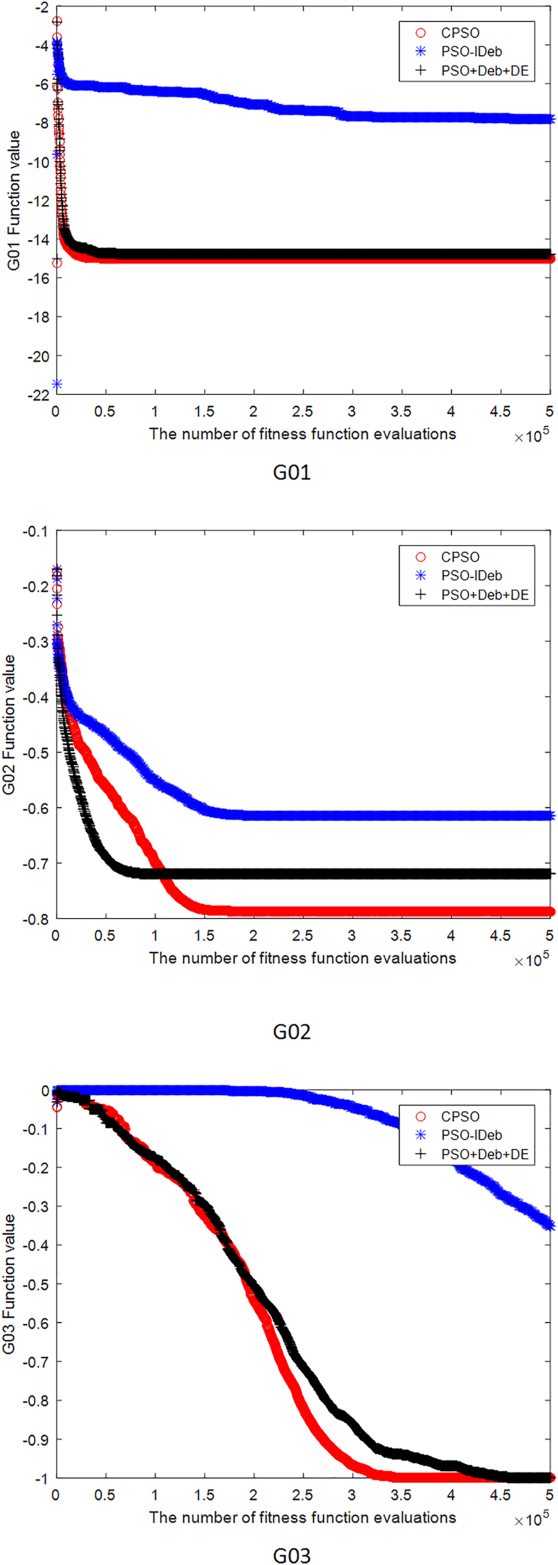
The mean convergence curves of function G01, G02, G03 for the three algorithms.

**Figure 3:**
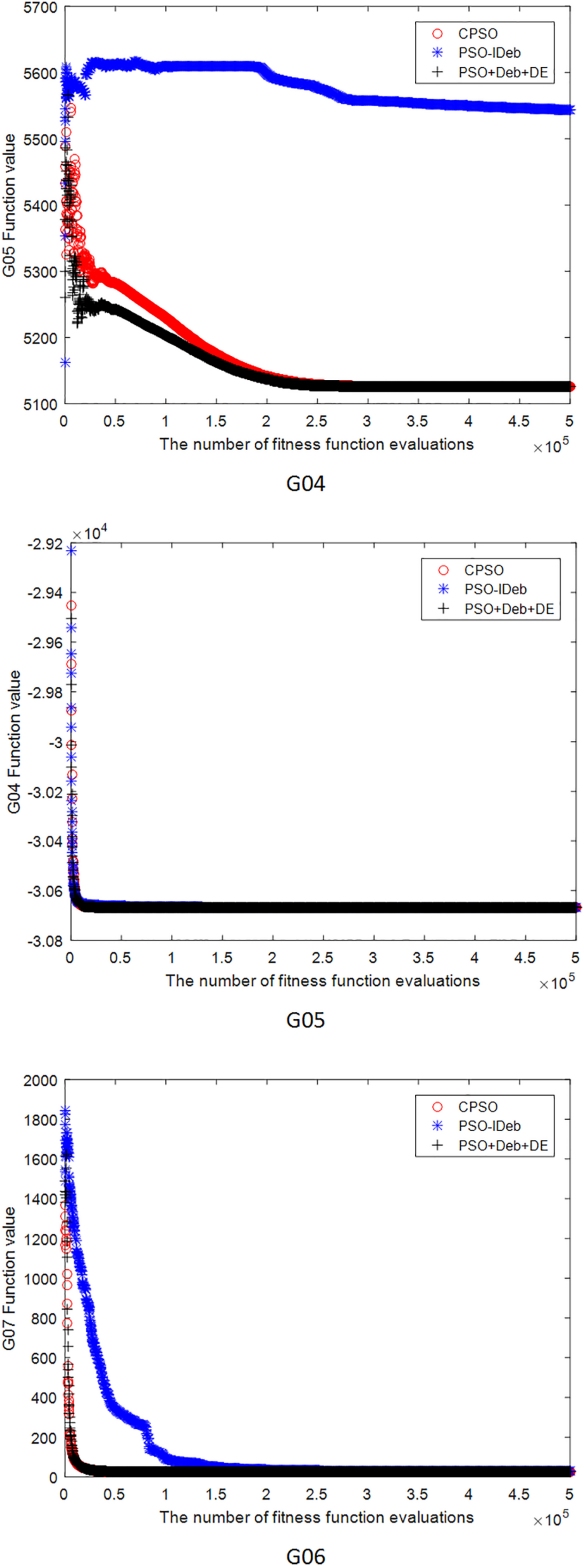
The mean convergence curves of function G04, G05, G06 for the three algorithms.

**Figure 4:**
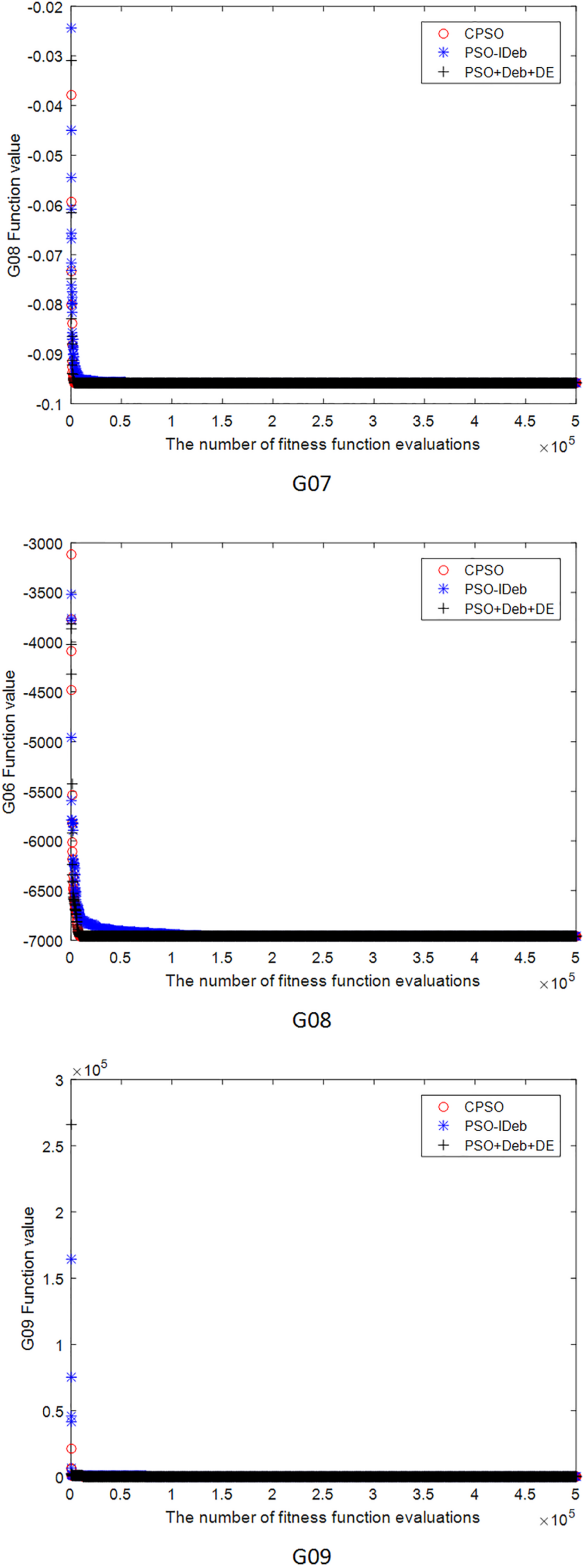
The mean convergence curves of function G07, G08, G09 for the three algorithms.

**Figure 5:**
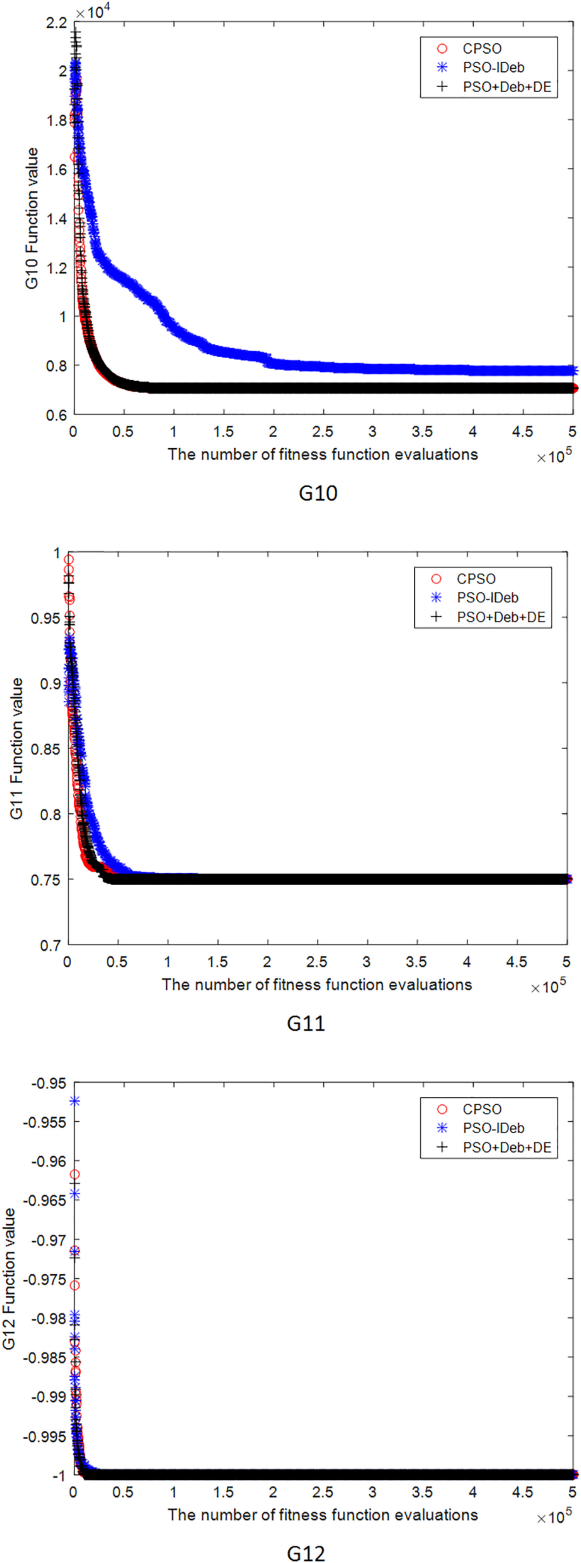
The mean convergence curves of function G10, G11, G12 for the three algorithms.

**Figure 6:**
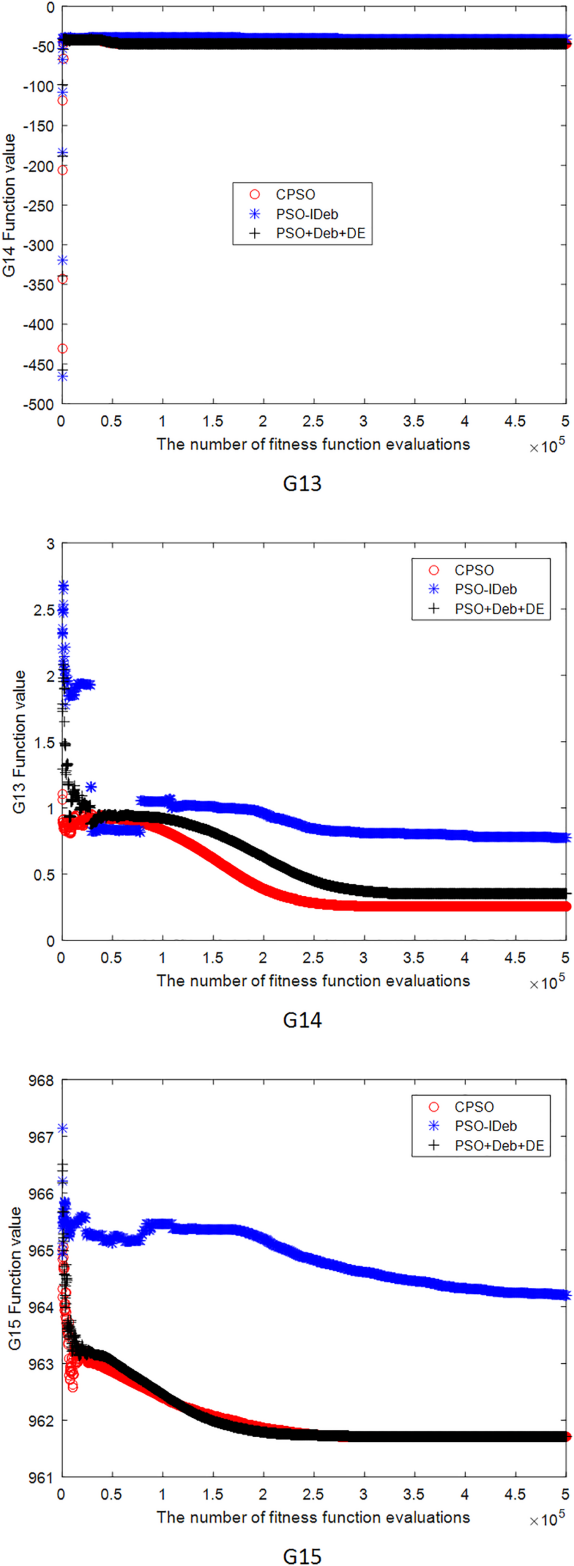
The mean convergence curves of function G13, G14, G15 for the three algorithms.

**Figure 7:**
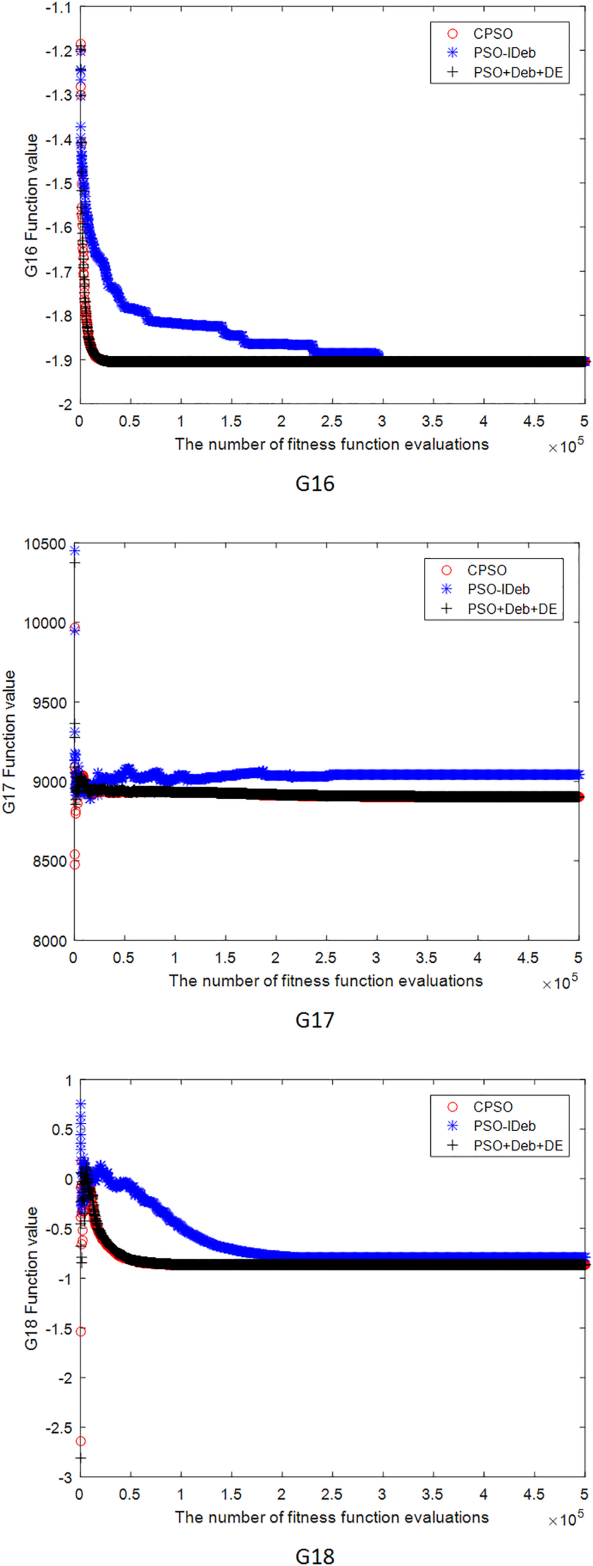
The mean convergence curves of function G16, G17, G18 for the three algorithms.

**Figure 8:**
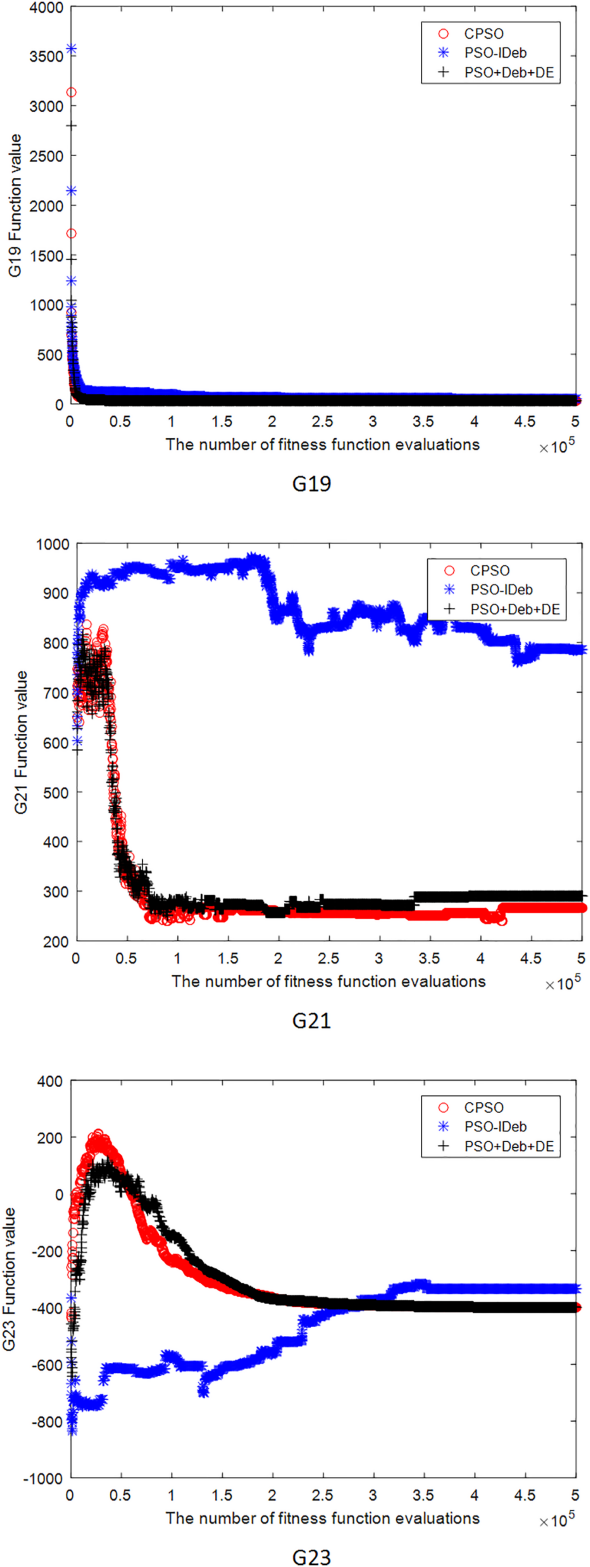
The mean convergence curves of function G19, G21, G23 for the three algorithms.

**Figure 9:**
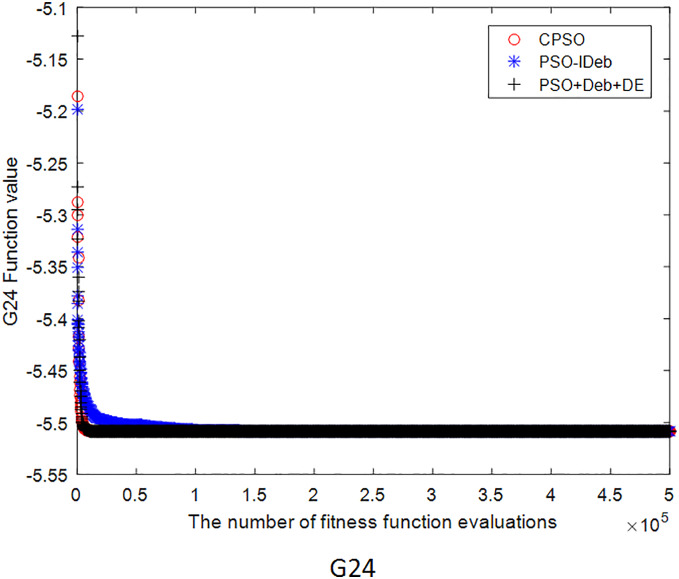
The mean convergence curves of function G24 for the three algorithms.

Figures 2–9 shows the mean convergence of the 22 functions for the three algorithms. The x-axis is the number of evaluations of the fitness function, and the y-axis is the mean of the function values for 25 runs. Note that for some functions (*e.g*., G01, G17, and G18), the function values may be smaller than the known optimal solution. These solutions appeared in the early stage of iteration and were therefore infeasible and invalid solutions that were eliminated during the later stage. Figures 2–9 shows that CPSO did not converge as fast as PSO+Deb+DE during the early stage for G02 and G05 but converged more rapidly during the later stage than the other two algorithms.

(2) Comparison of CPSO and HMPSO

As the DE strategy was used in both CPSO and HMPSO (Wang & Cai, 2009) to perform evolutionary operations on the optimal particle set, the numerical results of the two algorithms were compared to confirm the effectiveness of CPSO. The source program of the HMPSO algorithm was obtained from Yong Wang’s personal website. The same parameters were used to facilitate comparison. As the local search PSO algorithm is used for sub swarms in HMPSO, the parameters ${c_1}$, ${c_2}$, and w are not required. The other parameters were the same as those used in Section Test Functions and Parameter Settings.

Table 4 shows that the same numerical results were obtained using CPSO and HMPSO for seven functions (*i.e*., G01, G06, G11–12, G15–16, and G24). Both algorithms obtained the optimal solution for the remaining 15 functions, except HMPSO, which did not obtain the optimal solution for G13. The five worst solutions obtained by CPSO (*i.e*., for G03, G13, G17, G19, and G23) were better than those obtained by HMPSO, whereas the two worst solutions obtained by HMPSO (for G02 and G21) were better than those obtained by CPSO. Both algorithms converged to the same solution for the other functions. Both algorithms achieved the same mean value for nine functions (*i.e*., G04–05, G07–10, G14, G18, and G23). CPSO produced a better mean than HMPSO for four functions (*i.e*., G3, G13, G17, and G19), and HMPSO produced better mean values for the remaining two functions. A smaller standard deviation was obtained by using CPSO than by using HMPSO for 11 functions, not including G2, G8, G13, and G21. In particular, the standard deviation obtained by using CPSO for G18 and G19 was nearly nine orders of magnitude smaller than that of HMPSO. The Wilcoxon test results show that CPSO had the same performance as HMPSO on 18 functions, a better performance in three functions, and a worse performance in function G21. These results show a higher convergence and stability for CPSO than HMPSO in solving the 22 selected functions.

**Table 4:** Comparing CPSO with respect to HMPSO on 22 benchmark test functions.

Prob.	Algorithm	Best	Worst	Median	Mean (Wil Test)	Std
G01	CPSO	−1.500000E+01	−1.500000E+01	−1.500000E+01	−1.500000E+01	0
	HMPSO	−1.500000E+01	−1.500000E+01	−1.500000E+01	−1.500000E+01(=)	0
G02	CPSO	−8.036191E−01	−7.485572E−01	−7.948968E−01	−7.872680E−01	1.9431E−02
	HMPSO	−8.036191E−01	−7.930840E−01	−8.036180E−01	−8.028473E−01(+)	2.6784E−03
G03	CPSO	−1.000500E+00	−9.945233E−01	−1.000500E+00	−1.000261E+00	1.1953E−03
	HMPSO	−1.000500E+00	−9.902628E−01	−1.000500E+00	−1.000046E+00(=)	2.0501E−03
G04	CPSO	−3.066554E+04	−3.066554E+04	−3.066554E+04	−3.066554E+04	3.7130E−12
	HMPSO	−3.066554E+04	−3.066554E+04	−3.066554E+04	−3.066554E+04(=)	7.4260E−12
G05	CPSO	5.126497E+03	5.126497E+03	5.126497E+03	5.126497E+03	2.7847E−12
	HMPSO	5.126497E+03	5.126497E+03	5.126497E+03	5.126497E+03(=)	2.9529E−12
G06	CPSO	−6.961814E+03	−6.961814E+03	−6.961814E+03	−6.961814E+03	0
	HMPSO	−6.961814E+03	−6.961814E+03	−6.961814E+03	−6.961814E+03(=)	0
G07	CPSO	2.430621E+01	2.430621E+01	2.430621E+01	2.430621E+01	8.2685E−15
	HMPSO	2.430621E+01	2.430621E+01	2.430621E+01	2.430621E+01(=)	1.4025E−11
G08	CPSO	−9.582504E−02	−9.582504E−02	−9.582504E−02	−9.582504E−02	1.2981E−17
	HMPSO	−9.582504E−02	−9.582504E−02	−9.582504E−02	−9.582504E−02(=)	0
G09	CPSO	6.806301E+02	6.806301E+02	6.806301E+02	6.806301E+02	2.3206E−13
	HMPSO	6.806301E+02	6.806301E+02	6.806301E+02	6.806301E+02(=)	2.8892E−13
G10	CPSO	7.049248E+03	7.049248E+03	7.049248E+03	7.049248E+03	2.9646E−12
	HMPSO	7.049248E+03	7.049248E+03	7.049248E+03	7.049248E+03(=)	1.2125E−10
G11	CPSO	7.499000E−01	7.499000E−01	7.499000E−01	7.499000E−01	1.1331E−16
	HMPSO	7.499000E−01	7.499000E−01	7.499000E−01	7.499000E−01(=)	1.1331E−16
G12	CPSO	−1.000000E+00	−1.000000E+00	−1.000000E+00	−1.000000E+00	0
	HMPSO	−1.000000E+00	−1.000000E+00	−1.000000E+00	−1.000000E+00(=)	0
G13	CPSO	5.394151E−02	4.388026E−01	4.388026E−01	2.540693E−01	1.9624E−01
	HMPSO	6.622277E−02	4.601212E−01	2.988803E−01	2.932468E−01(=)	1.4041E−01
G14	CPSO	−4.776489E+01	−4.776489E+01	−4.776489E+01	−4.776489E+01	2.9008E−14
	HMPSO	−4.776489E+01	−4.776489E+01	−4.776489E+01	−4.776489E+01(=)	2.4911E−14
G15	CPSO	9.617150E+02	9.617150E+02	9.617150E+02	9.617150E+02	5.8016E−13
	HMPSO	9.617150E+02	9.617150E+02	9.617150E+02	9.617150E+02(=)	5.8016E−13
G16	CPSO	−1.905155E+00	−1.905155E+00	−1.905155E+00	−1.905155E+00	4.5325E−16
	HMPSO	−1.905155E+00	−1.905155E+00	−1.905155E+00	−1.905155E+00(=)	4.5325E−16
G17	CPSO	8.853534E+03	8.927979E+03	8.927592E+03	8.900967E+03	3.6308E+01
	HMPSO	8.853534E+03	8.945821E+03	8.927765E+03	8.905549E+03(=)	3.7560E+01
G18	CPSO	−8.660254E−01	−8.660254E−01	−8.660254E−01	−8.660254E−01	4.5325E−17
	HMPSO	−8.660254E−01	−8.660254E−01	−8.660254E−01	−8.660254E−01(=)	1.1413E−08
G19	CPSO	3.265559E+01	3.265559E+01	3.265559E+01	3.265559E+01	2.2328E−14
	HMPSO	3.265560E+01	3.265571E+01	3.265561E+01	3.265562E+01(+)	2.4444E−05
G21	CPSO	1.937245E+02	9.454397E+02	1.937245E+02	2.657074E+02	1.5449E+02
	HMPSO	1.937245E+02	3.247028E+02	1.937245E+02	2.356388E+02(−)	6.2357E+01
G23	CPSO	−4.000551E+02	−4.000550E+02	−4.000551E+02	−4.000551E+02	1.5844E−05
	HMPSO	−4.000551E+02	−4.000189E+02	−4.000551E+02	−4.000528E+02(+)	7.5429E−03
G24	CPSO	−5.508013E+00	−5.508013E+00	−5.508013E+00	−5.508013E+00	9.0649E−16
	HMPSO	−5.508013E+00	−5.508013E+00	−5.508013E+00	−5.508013E+00(=)	9.0649E−16
+/=/—		HMPSO:	3/18/1			

**Note:**

Best denotes the best solution; Worst denotes the worst solution; Median denotes the median value; Mean denotes the mean value; and Std denotes the standard deviation. Wil test denotes the Wilcoxon test result.

(3) Comparison of CPSO and other algorithms

To further illustrate its effectiveness, the CPSO algorithm was compared with other intelligent algorithms from the literature. The main parameters of each algorithm were set as follows: the number of evaluations of the fitness function was $2.4 \times {10^5}$, the mutation probability was 0.6, and the crossover probability was 0.8 in the GA algorithm (Mezura-Montes & Coello, 2005). The number of evaluations of the fitness function was $2.4 \times {10^5}$, and the correction rate was 0.8 in the ABC algorithm (Karaboga & Akay, 2011). The number of evaluations of the fitness function was $2.8 \times {10^5}$ in the CSHPSO algorithm (Yadav & Deep, 2014). The number of evaluations of the fitness function was $3.5 \times {10^5}$ in the PESO algorithm (Zavala, Aguirre & Diharce, 2005). The number of evaluations of the fitness function $\in$ $[1.06 \times {10^4},1.401 \times {10^5}]$, $F \in [0.9,1.0]$, and $CR \in [0.95,1]$ in the PSO-DE algorithm (Liu, Cai & Wang, 2010). Because most studies only discuss the test problems G01-G13, the performance of each algorithm on these 13 problems is analysed below.

The data in Table 5 are the best value and average value obtained by solving 13 test problems with six algorithms, and the best value obtained by each problem is expressed in bold (because the data retention digits used in each study are different, the comparison is based on the rounding result of the last digit). If the optimal value is the same, then all are bold. The best value and average value of G12 were successfully obtained by the six algorithms. However, for G02, G05 and G13, the performance of each algorithm decreased. Except for the average value of G02 obtained by the GA algorithm, the other best values were only solved by the CPSO algorithm, and the other algorithms were far from the real best solution; the GA algorithm did not obtain a feasible solution on G05 and G13. The PSO-DE, PESO and ABC algorithms obtained the best value and the best average value on seven, six and four problems, respectively. The CSHPSO algorithm only obtained the best value on G01, G08 and G12, but the best value and average value of the algorithm were essentially the same, which demonstrates that the stability of the CSHPSO algorithm is good, but the ability to jump out of local extremum needs to be improved. In the CPSO algorithm, except for the fact that the average value of G02 and G03 did not obtain the best solution, the other numerical results are among the best values. The CPSO algorithm had a better robustness and optimization effect than the other five algorithms in solving these 13 problems.

**Table 5:** Results obtained by the six algorithms on test problems G01–G13.

Prob.	Ind.	GA	ABC	CSHPSO	PESO	PSO-DE	CPSO
G01	Best	−14.440	−15.000	−15.0000	−15.00000	−15.00000	−15.0000000000
	Mean	−14.236	−15.000	−15.0000	−15.00000	−15.00000	−15.0000000000
G02	Best	−0.796231	−0.803598	−0.6900	−0.792608	−0.8036145	−0.8036191000
	Mean	−0.788588	−0.792412	−0.4970	−0.721749	−0.756678	−0.7872680328
G03	Best	−0.990	−1.000	−0.1030	−1.0005010	−1.0005010	−1.0005001000
	Mean	−0.976	−1.000	−0.1030	−1.0005006	−1.0005010	−1.0002609791
G04	Best	−30,626.053	−30,665.539	−30,700.0000	−30,665.538672	−30,665.5387	−30,665.5386717833
	Mean	−30,590.455	−30,665.539	−30,700.0000	−30,665.538672	−30,665.5387	−30,665.5386717833
G05	Best	NF	5,126.484	5,290.0000	5,126.484154	--	5,126.4967140071
	Mean	NF	5,185.714	5,290.0000	5,129.178298	--	5,126.4967140071
G06	Best	−6,952.472	−6,961.814	−6,960.0000	−6,961.813876	−6,961.81388	−6,961.81387558023
	Mean	−6,872.204	−6,961.813	−6,960.0000	−6,961.813876	−6,961.81388	−6,961.8138755802
G07	Best	31.097	24.330	24.3000	24.306921	24.3062091	24.3062090682
	Mean	34.980	24.473	24.3000	24.371253	24.3062100	24.3062090682
G08	Best	−0.095825	−0.095825	−0.0958	−0.095825	−0.09582594	−0.0958250414
	Mean	−0.095799	−0.095825	−0.0958	−0.095825	−0.09582594	−0.0958250414
G09	Best	685.994	680.634	681.0000	680.630057	680.63006	680.6300573744
	Mean	692.064	680.640	681.0000	680.630057	680.63006	680.6300573744
G10	Best	9,079.770	7,053.904	7,050.0000	7,049.459452	7,049.248021	7,049.2480205287
	Mean	10,003.225	7,224.407	7,050.0000	7,099.101385	7,049.248038	7,049.2480205287
G11	Best	0.750	0.750	0.7500	0.749000	0.749999	0.7499000000
	Mean	0.750	0.750	0.7500	0.749000	0.749999	0.7499000000
G12	Best	−1.000	−1.000	−1.0000	−1.000000	−1.000000	−1.0000000000
	Mean	−1.000	−1.000	−1.0000	−1.000000	−1.000000	−1.0000000000
G13	Best	0.134057	0.760	0.4390	0.8.1498	--	0.0539415140
	Mean	NF	0.968	0.4390	0.626881	--	0.2540692828

**Note:**

Where, “NF” is no feasible solution; “--” is not involved in the literature.

## Constrained real-world optimization

In this section, we select three real-world constraint optimization problems to test the CPSO algorithm (Kumar et al., 2020). We compare it with the top three algorithms of IEEE Evolutionary Computing Conference (CEC2020) and Genetic and Evolutionary Computation Congress (GECCO2020): Self-Adaptive Spherical Search Algorithm (SASS) (Kumar, Das & Zelinka, 2020), LSHADE for Constrained Optimization with Lévy Flight (COLSHADE) (Gurrola-Ramos, Hernandez-Aguirre & Dalmau-Cedeno, 2020), and Modified Covariance Matrix Adaptation Evolution Strategy (sCMAgES) (Kumar, Das & Zelinka, 2020).

(1) Optimal operation of alkylation unit problem

The main aim of this problem is to maximize the octane number of olefin feed in the presence of acid. The objective function is defined as an alkylating product. The problem is formulated as follows:

$\matrix{ {\min } \hfill & {f(x) = - 0.035{x_1}{x_6} - 1.715{x_1} - 10.0{x_2} - 4.0565{x_3} + 0.063{x_3}{x_5}} \hfill \cr {s.t.} \hfill & {{\rm }{g_1}(x) = 0.0059553571x_6^2{x_1} + 0.88392857{x_3} - 0.1175625{x_6}{x_1} - {x_1} \le 0} \hfill \cr {} \hfill & {{g_2}(x) = 1.1088{x_1} + 0.1303533{x_1}{x_6} - 0.0066033{x_1}x_6^2 - {x_3} \le 0} \hfill \cr {} \hfill & {{g_3}(x) = 6.66173269x_6^2 + 56.596669{x_4} + 172.39878{x_5} - 1000 - 191.20592{x_6} \le 0} \hfill \cr {} \hfill & {{g_4}(x) = 1.08702{x_6} - 0.03762x_6^2 + 0.32175{x_4} + 56.85075 - {x_5} \le 0} \hfill \cr {} \hfill & {{g_5}(x) = 0.006198{x_7}{x_4}{x_3} + 2562.3121{x_2} - 25.125634{x_2}{x_4} - {x_3}{x_4} \le 0} \hfill \cr {} \hfill & {{g_6}(x) = 161.18996{x_4}{x_3} + 5000.0{x_2}{x_4} - 489510.0{x_2} - {x_3}{x_4}{x_7} \le 0} \hfill \cr {} \hfill & {{g_7}(x) = 0.33{x_7} + 44.333333 - {x_5} \le 0} \hfill \cr {} \hfill & {{g_8}(x) = 0.022556{x_5} - 1.0 - 0.007595{x_7} \le 0} \hfill \cr {} \hfill & {{g_9}(x) = 0.00061{x_3} - 1.0 - 0.0005{x_1} \le 0} \hfill \cr {} \hfill & {{g_{10}}(x) = 0.8196721{x_1} - {x_3} + 0.819672 \le 0} \hfill \cr {} \hfill & {{g_{11}}(x) = 24500.0{x_2} - 250.0{x_2}{x_4} - {x_3}{x_4} \le 0} \hfill \cr {} \hfill & {{g_{12}}(x) = 1020.4082{x_4}{x_2} + 1.2244898{x_3}{x_4} - 100000{x_2} \le 0} \hfill \cr {} \hfill & {{g_{13}}(x) = 6.25{x_1}{x_6} + 6.25{x_1} - 7.625{x_3} - 100000 \le 0} \hfill \cr {} \hfill & {{g_{14}}(x) = 1.22{x_3} - {x_6}{x_1} - {x_1} + 1.0 \le 0} \hfill \cr {} \hfill & {1000 \le {x_1} \le 2000,0 \le {x_2} \le 100} \hfill \cr {} \hfill & {2000 \le {x_3} \le 4000,0 \le {x_4} \le 100} \hfill \cr {} \hfill & {0 \le {x_5} \le 100,0 \le {x_6} \le 20} \hfill \cr {} \hfill & {0 \le {x_7} \le 200} \hfill \cr } {\rm }$

Table 6 shows the result of the optimal operation of the alkylation unit. All algorithms obtained a feasible solution to this problem. The best values obtained by CPSO and COLSHADH reached −4,529.1197. The worst and mean values found by CPSO are smaller than the best results obtained by the other algorithms. The best std value was obtained by SASS; however, in the process of searching for the best value, the results of this algorithm are not ideal, indicating that the algorithm became trapped in a local extreme value on this problem. Therefore, compared with other algorithms, CPSO is more stable for solving this problem.

**Table 6:** The results of optimal operation of alkylation unit problem.

*Prob*.	CPSO	SASS	sCMAgES	COLSHADH
Best	−4,529.1197	−142.7193	−4,527.7659	−4,529.1197
Worst	−4,529.1189	−142.7193	−3,536.3552	−3,716.9077
Median	−4,529.1196	−142.7193	−4,427.2452	−4,529.1197
Mean	−4,529.1196	−142.7193	−4,324.9110	−4,366.6773
Std	2.3407E−04	2.1985e−05	269.2210	324.8848
Feasible rate%	100	100	100	100

(2) Weight minimization of a speed reducer problem

This problem involves the design of a speed reducer for a small aircraft engine. The resulting optimization problem is formulated as follows:

$\matrix{ {\min } \hfill & \matrix{f(x) = 0.7854x_2^2{x_1}(14.9334{x_3} - 43.0934 + 3.3333x_3^2) + 0.7854({x_5}x_7^2 + {x_4}x_6^2) \hfill \cr - 1.508(x_7^2 + x_6^2) + 7.447(x_7^3 + x_6^3) \hfill} \hfill \cr {s.t.} \hfill & {{g_1}(x) = - {x_1}x_2^2{x_3} + 27 \le 0} \hfill \cr {} \hfill & {{g_2}(x) = - {x_1}x_2^2x_3^2 + 397.5 \le 0} \hfill \cr {} \hfill & {{g_3}(x) = - {x_2}x_6^4{x_3}x_4^{ - 3} + 1.93 \le 0} \hfill \cr {} \hfill & {{g_4}(x) = - {x_2}x_7^4{x_3}x_5^{ - 3} + 1.93 \le 0} \hfill \cr {} \hfill & {{g_1}(x) = 10x_6^{ - 3}\sqrt {16.91 \times {{10}^6} + {{(745{x_4}x_2^{ - 1}x_3^{ - 1})}^2}} - 1100 \le 0} \hfill \cr {} \hfill & {{g_1}(x) = 10x_7^{ - 3}\sqrt {157.5 \times {{10}^6} + {{(745{x_5}x_2^{ - 1}x_3^{ - 1})}^2}} - 850 \le 0} \hfill \cr {} \hfill & {{g_1}(x) = {x_2}{x_3} - 40 \le 0} \hfill \cr {} \hfill & {{g_1}(x) = - {x_1}x_2^{ - 1} + 5 \le 0} \hfill \cr {} \hfill & {{g_1}(x) = {x_1}x_2^{ - 1} - 12 \le 0} \hfill \cr {} \hfill & {{g_1}(x) = 15{x_6} - {x_4} + 1.9 \le 0} \hfill \cr {} \hfill & {{g_1}(x) = 1.1{x_7} - {x_5} + 1.9 \le 0} \hfill \cr {} \hfill & {0.7 \le {x_2} \le 0.8,17 \le {x_3} \le 28,2.6 \le {x_1} \le 3.6} \hfill \cr {} \hfill & {5 \le {x_7} \le 5.5,7.3 \le {x_5},{x_4} \le 8.3,2.9 \le {x_6} \le 3.9} \hfill \cr }$

Table 7 reveals the results of the weight minimization of a speed reducer problem. For this problem, all algorithms obtained similar results, and the stability of SASS and COLSHADH is slightly better. The std value of CPSO also reached ${10^{ - 8}}$, which indicates that the CPSO algorithm is stable and effective in solving such problems.

**Table 7:** The results of weight minimization of a speed reduce problem.

*Prob*.	CPSO	SASS	sCMAgES	COLSHADH
Best	2,994.4245	2,994.4245	2,994.4245	2,994.4245
Worst	2,994.4245	2,994.4245	2,994.4245	2,994.4245
Median	2,994.4245	2,994.4245	2,994.4245	2,994.4245
Mean	2,994.4245	2,994.4245	2,994.4244	2,994.4245
Std	6.1883E−08	4.6412e−13	2.7723E−12	4.5475E−13
Feasible rate%	100	100	100	100

(3) Planetary gear train design optimization problem

The main objective of this problem is to minimize the maximum errors in the gear ratio, which is used in automobiles. To minimize the maximum error, the total number of gear teeth is calculated for an automatic planetary transmission system. The problem is formulated as follows:

$\matrix{ {\min } & {f(x) = \max \left| {{i_k} - {i_{0k}}} \right|,k = \{ 1,2,...,R\} } \cr {} & {{i_1} = \displaystyle{{{N_6}} \over {{N_4}}},{i_{01}} = 3.11,{i_2} = \displaystyle{{{N_6}({N_1}{N_3} + {N_2}{N_4})} \over {{N_1}{N_3}({N_6} - {N_4})}},{i_{0R}} = - 3.11} \cr {} & {{I_R} = - \displaystyle{{{N_2}{N_6}} \over {{N_1}{N_3}}},{i_{02}} = 1.84,x = \{ p,{N_6},{N_5},{N_4},{N_3},{N_2},{N_1},{m_2},{m_1}\} } \cr {s.t.} & {{g_1}(x) = {m_3}({N_6} + 2.5) - {D_{\max }} \le 0} \cr {} & {{g_2}(x) = {m_1}({N_1} + {N_2}) + {m_1}({N_2} + 2) - {D_{\max }} \le 0} \cr {} & {{g_3}(x) = {m_3}({N_4} + {N_5}) + {m_3}({N_2} + 2) - {D_{\max }} \le 0} \cr {} \hfill & {{g_4}(x) = \left| {{m_1}({N_1} + {N_2}) - {m_3}({N_6} - {N_3})} \right| - {m_1} - {m_3} \le 0} \cr {} & {{g_5}(x) = - ({N_1} + {N_2})\sin ({\pi \mathord{\left/ {\vphantom {\pi p}} \right. } p}) + {N_2} + 2 + {\delta _{22}} \le 0} \cr {} & {{g_6}(x) = - ({N_6} - {N_3})\sin ({\pi \mathord{\left/ {\vphantom {\pi p}} \right. } p}) + {N_3} + 2 + {\delta _{33}} \le 0} \cr {} & {{g_7}(x) = - ({N_4} + {N_5})\sin ({\pi \mathord{\left/ {\vphantom {\pi p}} \right. } p}) + {N_3} + 2 + {\delta _{33}} \le 0} \cr {} & {{g_8}(x) = {{({N_3} + {N_5} + 2 + {\delta _{35}})}^2} - {{({N_6} - {N_3})}^2} - {{({N_4} + {N_5})}^2}} \cr {} & {{\rm } + 2({N_6} - {N_3})({N_4} + {N_5})\cos \bigg(\displaystyle{{2\pi } \over p} - \beta \bigg) \le 0} \cr {} & {{g_9}(x) = {N_4} - {N_6} + 2{N_5} + 2{\delta _{56}} + 4 \le 0} \cr {} & {{g_{10}}(x) = 2{N_3} - {N_6} + {N_4} + 2{\delta _{34}} + 4 \le 0} \cr {} & {{h_1}(x) = \displaystyle{{{N_6} - {N_4}} \over p} = {\rm integer}} \cr {} & {{\delta _{22}} = {\delta _{33}} = {\delta _{55}} = {\delta _{35}} = {\delta _{56}} = 0.5} \cr {} & {\beta = \displaystyle{{{{\cos }^{ - 1}}({{({N_4} + {N_5})}^2} + {{({N_6} - {N_3})}^2} - {{({N_3} + {N_5})}^2})} \over {2({N_6} - {N_3})({N_4} + {N_5})}},{D_{\max }} = 220} \cr {} & {p = (3,4,5)} \cr {} & {{m_1} = (1.75,2.0,2.25,2.5,2.75,3.0)} \cr {} & {{m_3} = (1.75,2.0,2.25,2.5,2.75,3.0)} \cr {} & {17 \le {N_1} \le 96,14 \le {N_2} \le 54,14 \le {N_3} \le 51} \cr {} & {17 \le {N_4} \le 46,14 \le {N_5} \le 51,48 \le {N_6} \le 124} \cr {} & {{N_i} = {\rm integer}} \cr }$

Table 8 reports the results on the planetary gear train design optimization problem. CPSO outperformed SASS, COLSHADE and sCMAgES. CPSO yielded a best value of 0.5256, whereas the best values for SASS, COLSHADE and sCMAgES are 0.5258, 0.5258 and 0.5260, respectively. CPSO has a significantly small worst std value, which demonstrates the stability of the CPSO algorithm in solving such problems. All data in Table 8 indicate that CPSO is superior to its competitors in this problem. The results found by CPSO are superior to those of the other three algorithms.

**Table 8:** The results of planetary gear train design optimization problem.

*Prob*.	CPSO	SASS	sCMAgES	COLSHADH
Best	0.5256	0.5258	0.5260	0.5258
Worst	0.5406	3.5217	0.5438	0.7467
Median	0.5300	0.6456	0.5300	0.5300
Mean	0.5317	1.0015	0.5308	0.5410
Std	3.9412E−03	7.2518E−01	4.3484E−03	4.2573E−02
Feasible rate%	100	80	100	100

## Conclusions

A PSO algorithm based on an improved Deb criterion, referred to as CPSO, was proposed for solving COPs. We developed a strategy for updating the current swarm using an ‘excellent’ infeasible solution set to address incomplete utilization of infeasible information in the existing Deb criterion, and the DE strategy was used to update the optimal particle set to improve the global convergence of the algorithm. We verified the effectiveness of the proposed algorithm by comparing numerical results obtained by the CPSO algorithm to those obtained using PSO+Deb+DE and PSO+Ideb. The numerical results show that PSO incorporated with Deb and DE effectively solved 22 test functions from CEC2006. Finally, comparing the performance of the CPSO algorithm to that of other algorithms demonstrated the effectiveness and stability of the proposed algorithm for solving the considered 13 functions and three real-world constraint optimization problems.

In future research, we will mainly focus on two aspects. First, effective operation strategies will be designed to solve more complex constrained optimization problems, such as multiobjective constrained optimization problems and mixed-integer constrained optimization problems. Second, more effective and targeted operators will be designed to form an operator pool, enabling the CPSO algorithm to solve more real-world problems such as data clustering, engineering optimization and image segmentation.

## Supplemental Information

10.7717/peerj-cs.1178/supp-1 Supplemental Information 1 Matlab program for CPSO.

## Additional Information and Declarations

### Data Availability

The following information was supplied regarding data availability: The Matlab code is available in the Supplemental Files.
